# Zembrin^®^ Mitigates Reserpine-Induced Motor Dysfunction and Oxidative Stress in Parkinson’s Disease: In Vivo and In Silico Analyses

**DOI:** 10.3390/molecules31132369

**Published:** 2026-07-05

**Authors:** Keagile Lepule, Maxleene Sandasi, Elliasu Salifu, Alvaro Viljoen

**Affiliations:** 1 Department of Pharmaceutical Sciences, Tshwane University of Technology, Private Bag X680, Pretoria 0001, South Africa; keagilebasetsana@gmail.com (K.L.); sandasim@tut.ac.za (M.S.); salifuey@tut.ac.za (E.S.); 2 SAMRC Herbal Drugs Research Unit, Faculty of Science, Tshwane University of Technology, Pretoria 0001, South Africa

**Keywords:** locomotion, *Mesembryanthemum tortuosum*, molecular docking, Parkinson’s disease, reserpine, zebrafish larvae

## Abstract

Parkinson’s disease (PD), impacting millions worldwide, leads to motor deficits and various non-motor symptoms. Although there is no cure, treatment primarily involves dopamine replacement therapy, especially L-dopa for motor symptoms, and additional drugs are required to address non-motor effects. This underscores the increasing demand for dual-acting drugs that can effectively target both symptom types in PD. This study explored the potential effects of a standardised *Mesembryanthemum tortuosum* extract, Zembrin^®^, in treating PD, utilising in vivo and in silico models. Zebrafish larvae were subjected to pre-treatment with reserpine, followed by exposure to Zembrin^®^, with selegiline and L-dopa as positive controls. The in vivo component of this study monitored locomotion and oxidative stress, while the in silico component identified potential drug targets for the treatment of PD. Reserpine induced hypolocomotion and oxidative stress in zebrafish larvae, and Zembrin^®^ (12.5 µg/mL) effectively enhanced locomotion and reduced oxidative stress. The molecular docking, molecular dynamics simulations, and binding free energy calculations revealed that four mesembrine alkaloids (mesembranol, mesembrenol, mesembrenone, and mesembrine) form stable and energetically favourable complexes with monoamine oxidase B (MAO-B) and dopamine transporter (DAT), which are significant targets for addressing both the motor and non-motor effects of PD.

## 1. Introduction

Parkinson’s disease (PD) is one of the most prevalent neurodegenerative diseases in the world [1]. In 2015, the Global Burden of Disease, Injuries and Risk Factors Studies reported that PD had the fastest-growing prevalence, disability, and deaths [2]. Furthermore, a more recent study projected that the global number of PD cases will reach 25.2 million by 2050, a 112-fold increase in global cases [3]. Parkinson’s disease is a chronic condition that is caused by the loss of dopaminergic neurons in the substantia nigra pars compacta (SNpc) region of the brain [4]. The four cardinal clinical symptoms of PD are tremor, rigidity, akinesia (lack of movement) or bradykinesia, and postural instability (balance dysfunction) [4,5]. Parkinson’s disease not only manifests cardinal motor symptoms but also includes a range of non-motor symptoms, such as sleep disorders, depression, anxiety, psychosis, cognitive decline, memory loss, and dementia, indicating a broader disease pathology beyond the nigrostriatal system [5,6,7]. While PD has no cure, treatment primarily involves dopamine replacement therapy, specifically L-dopa. Treatment with L-dopa significantly benefits most patients, enhancing daily activities, independence, employability, and survival. However, prolonged use of L-dopa can result in complications, such as motor fluctuations, dyskinesias, and neuropsychiatric issues, including depression and cognitive deficits [4]. There is an augmented demand for new treatments capable of addressing both motor and non-motor symptoms of PD. In recent years, there has been an increasing interest in non-pharmacological interventions (NPIs) to treat a wide range of medical conditions, particularly those without a cure [8,9].

Plant-derived medicines have been classified under NPIs and have long been used to treat various conditions, and they continue to provide new chemical entities that are useful medicines [10]. Plant-derived medicines range from concoctions, teas, or concentrated plant extracts, often without a specific active ingredient. Recently, there has been a global spike in the use of medicinal plants for promoting health and to some extent, these plant-derived medicines are prescribed in developed countries [8]. An example of a plant-derived herbal product is Zembrin^®^, from *Mesembryanthemum tortuosum*, an indigenous plant to Southern Africa. Zembrin^®^ has been marketed for the treatment of moderate stress and anxiety (www.zembrin.com). Additionally, several studies have demonstrated that Zembrin^®^ is safe and has antidepressant, anxiolytic, and mood-enhancing properties, established using in vitro, in vivo, and ex vivo models [11,12,13,14,15], as well as clinical trials involving healthy participants [16,17]. More recently, studies have shown that *M. tortuosum* extracts have neuroprotective effects that could benefit neurodegenerative diseases, such as PD [18,19,20]. Previously, we reported that Zembrin^®^ ameliorated locomotor impairment and oxidative stress in a 6-OHDA-induced zebrafish model of PD. To further validate its therapeutic potential, the present study employs a reserpine-induced model, which represents a distinct pathogenic mechanism involving monoamine depletion. Furthermore, computational approaches, including molecular docking, were used to explore putative molecular targets and provide mechanistic insights into the observed neuroprotective effects of Zembrin^®^.

Modelling PD poses a unique challenge to highlight both motor and non-motor symptoms. The motor aspects are primarily related to disturbed dopaminergic neurotransmission, whereas the non-motor features show that neurotransmitter dysfunction is not exclusive to the dopaminergic pathway [5,6,7]. Reserpine induces PD by blocking vesicular monoamine transporter 2 (VMAT2), thereby impairing the neurotransmission of dopamine, noradrenaline, and serotonin [21,22]. Although the use of reserpine in PD modelling has declined over time [23], various in vivo studies show that administration of reserpine induces severe motor deficits and oxidative stress consistent with key pathological and behavioural aspects of PD [22,24,25,26]. This, therefore, suggests that reserpine models may be used as a valuable tool in preclinical studies on motor and non-motor symptoms of PD.

For over two decades, zebrafish (*Danio rerio*) have emerged as a valuable model for investigating neurological disorders due to their 80% physiological similarity to mammals, including brain structure and neurotransmission [27,28]. Early developments are crucial as dopaminergic neurons appear around 18–19 h post-fertilisation (hpf), and by 4–5 days post-fertilisation (dpf), neuronal connections are formed with active neurotransmitter release [29]. Furthermore, at 3 dpf, zebrafish larvae develop a blood–brain barrier (BBB) similar to that of higher primates [30]. Their sensitivity to psychoactive drugs also contributes to their usefulness in research [27,31].

Various studies have reported that mesembrine alkaloids possess psychomodulatory effects and have been linked to inhibition of serotonin reuptake transport (SERT) and phosphodiesterase-4 (PDE4) [11,32]. However, the ability of mesembrine alkaloids to modulate drug targets implicated in the treatment of PD has not been fully explored. Therefore, in the current study, four mesembrine alkaloids were subjected to in silico computational studies to identify and explore molecular docking for potential drug targets for the treatment of PD. Additionally, the activity of commercial standardised *M. tortuosum* extract, Zembrin^®^, were further explored using a reserpine model for PD in zebrafish larvae. Hence, the aim of this study was to evaluate the effects of commercial standardised *M. tortuosum* extract, Zembrin^®^, on PD using in vivo and in silico models.

## 2. Results

### 2.1. Phytochemical Profiling

**Figure 1:**
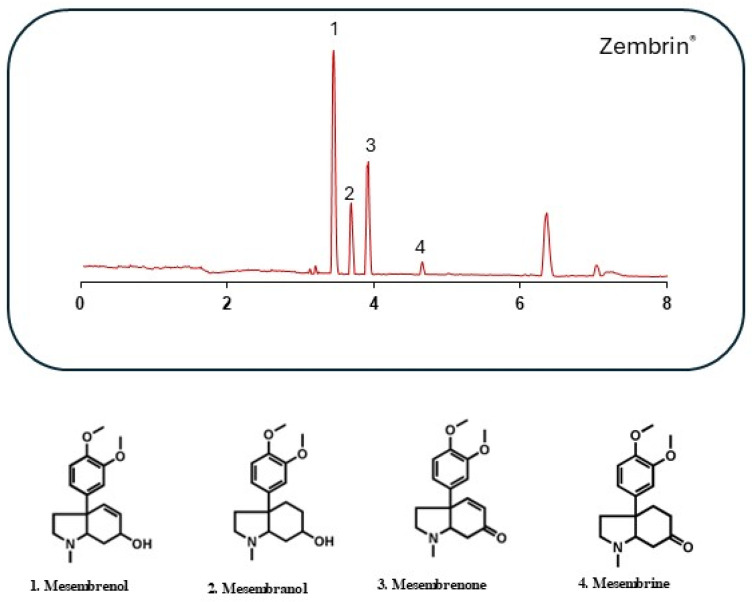
The phytochemical profile of mesembrine-type alkaloids found in the standardised *M. tortuosum* extract, Zembrin^®^.

The phytochemical profiling of Zembrin^®^ showed the presence of mesembrenol, mesembranol, mesembrenone, and mesembrine, and each of the alkaloids identified has been reported in previous studies [15,33,34] (Figure 1).

### 2.2. The Effect of Zembrin^®^ on Reserpine-Induced Locomotor and Oxidative Stress Alterations

A preliminary maximum tolerable concentration (MTC) determination assay showed that Zembrin^®^ did not induce mortality at the tested concentrations (100–500 µg/mL). However, decreased swimming behaviour was observed at higher concentrations; therefore, subsequent efficacy experiments were conducted at lower concentrations (12.5, 25, and 50 µg/mL).

**Figure 2:**
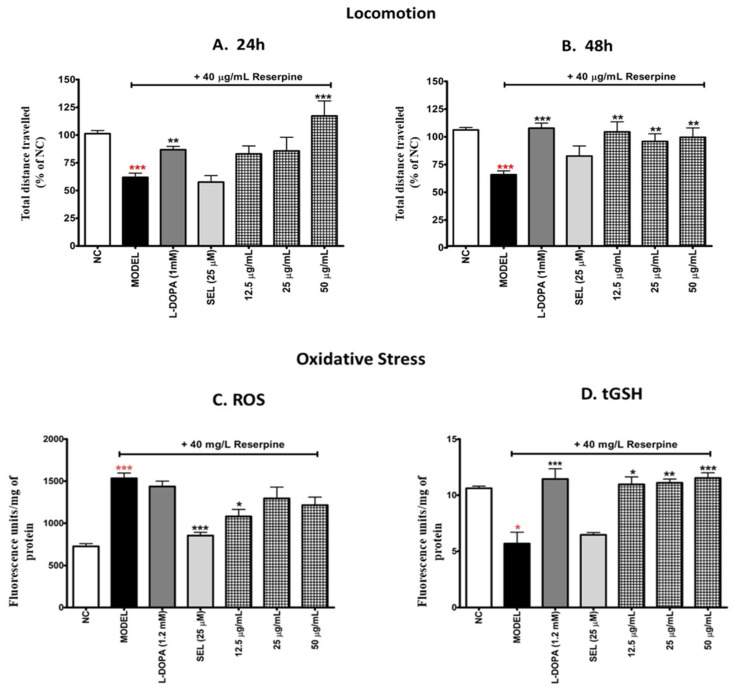
A depiction of locomotor activity and oxidative stress parameters in zebrafish larvae pretreated with reserpine, followed by treatment with Zembrin^®^ at different concentrations, and the positive controls (L-dopa and selegiline). Locomotion was monitored as distance travelled at 24 h (**A**) and 48 h (**B**). Oxidative stress was monitored as a measure of ROS (**C**) and tGSH (**D**). NC—negative control, SEL—selegiline treated, and MODEL—reserpine treated. Significant difference between the treatments and the model groups, * *p* < 0.05, ** *p* < 0.01, and *** *p* < 0.001. Significant difference between the models and the negative control (NC), * *p* < 0.05 and *** *p* < 0.001.

The reserpine-treated larvae displayed over 40% reduction in locomotion after 24 h and 48 h ([D = 140; *p* < 0.001] and [D = 144.7; *p* < 0.001], respectively) compared with the negative control (Figure 2A,B). This suggests that reserpine treatment mimics the PD behavioural repertoire of reduced locomotion. L-dopa-treated larvae demonstrated a reversal of reserpine-induced locomotor deficits at 24 h [D = −61; *p* < 0.01] and 48 h [D = −130.0; *p* < 0.001] (Figure 2A,B). Selegiline treatment improved reserpine-induced locomotor deficits by 17% [D = −25.83; *p* > 0.05] after 48 h only (Figure 2B); however, the results were not statistically significant, while no improvement was observed after 24 h (Figure 2A). After 24 h exposure to Zembrin^®^, larvae treated with the concentration of 50 µg/mL showed significant improvement in locomotor deficits, surpassing the negative control [D = −131.2; *p* < 0.001] (Figure 2A). At 48 h, Zembrin^®^ markedly improved locomotion at all the tested concentrations (12.5, 25, 50 µg/mL) [D = −106.0; D = −92.94; D = 101.1; *p* < 0.01], performing better than selegiline (Figure 2B).

Compared with the negative control, larvae treated with reserpine exhibited a 111% increase in ROS production [D = −95.00; *p* < 0.001] (Figure 2C). L-dopa improved reserpine-induced ROS by only 6% [D = 8.587; *p* > 0.05], whereas selegiline reduced ROS by 44% [D = 80.00; *p* > 0.001], thus outperforming L-dopa. Although all the tested concentrations of Zembrin^®^ reduced reserpine-induced ROS generation, the lowest concentration (12.5 µg/mL) displayed a 30% significant reduction [D = 51.15; *p* < 0.05] in ROS content (Figure 2C).

The tGSH content decreased by 47% due to exposure to reserpine in the treatment regimen [D = 66.40; *p* < 0.05] (Figure 2D). L-dopa was able to reverse the effects by increasing the tGSH content by two-fold [D = −95.01; *p* < 0.001] (Figure 2D). Although not statistically significant, selegiline improved tGSH by at least 10%. Similarly, all the tested concentrations of Zembrin^®^ significantly increased tGSH content ([D = −67.18; *p* < 0.05], [D = −78.52; *p* < 0.01] and [D = −81.28; *p* < 0.001]), performing better than the positive control selegiline (Figure 2D).

In summary, in the behavioural assay, the activity of selegiline was surpassed by Zembrin^®^ at 50 µg/mL, demonstrating superiority in improving reserpine-induced locomotor deficits at both time points (24 h and 48 h). In the ROS assay, selegiline displayed superior activity in scavenging ROS induced by reserpine treatment. Moreover, Zembrin^®^ demonstrated superior activity in the tGSH assay at all the tested concentrations, as evidenced by its ability to substantially reverse the decrease in tGSH that was induced by reserpine. Although selegiline showed limited effects in the locomotor assay and tGSH levels, it reduced ROS effectively, highlighting limitations of current therapies. Clinically, selegiline is not used as monotherapy but in combination with L-dopa to manage motor symptoms and dyskinesia, as it replenishes dopaminergic transmission. The modest locomotor response is, therefore, consistent with its clinical profile, as L-Dopa remains the primary treatment for motor deficits consistent with our study. The choice to use selegiline as a positive control was, therefore, based on its MAO-B inhibitory activity, a characteristic shared with mesembrine alkaloids, allowing mechanistic comparison.

### 2.3. Molecular Docking Analysis

The molecular docking analysis was conducted to explore the potential interactions between mesembrine-type alkaloids from Zembrin^®^ and selected protein targets implicated in the pathogenesis of PD (see Supplementary Table S2). For this study, docking scores ranging from approximately −6.7 to −8.7 kcal/mol were obtained, suggesting moderate to strong binding affinities between the investigated alkaloids and several PD-related molecular targets (Table 1). In binding energy analysis, more negative binding energy values are indicative of stronger and more favourable ligand–receptor interactions.

Among the evaluated targets, strong binding interactions were observed with MAO-B, particularly for mesembranol and mesembrenone, which exhibited estimated docking scores of −8.7 kcal/mol (Table 1). The enzyme MAO-B is a mitochondrial enzyme responsible for the oxidative metabolism of dopamine, a process that produces hydrogen peroxide and other reactive oxygen species that contribute to oxidative stress and neuronal damage in PD [35]. Pharmacological inhibition of MAO-B is, therefore, a well-established therapeutic strategy in PD management, with drugs such as selegiline and rasagiline widely used to inhibit the metabolism of monoamine neurotransmitters and reduce oxidative stress; hence, they are regarded as neuroprotective [36]. The strong binding affinity observed supports earlier experimental findings suggesting that Zembrin^®^ may possess MAO-B inhibitory activity and provides a plausible mechanistic explanation for the reduction in ROS and increased glutathione levels observed in the zebrafish model.

In addition to MAO-B, the mesembrine alkaloids demonstrated favourable binding interactions with the DAT (Table 1). The dopamine transporter plays a central role in regulating dopaminergic neurotransmission by mediating the reuptake of dopamine from the synaptic cleft into presynaptic neurons [37]. Compounds that interact with this transporter can enhance dopaminergic signalling by modulating dopamine availability in synaptic spaces. The relatively strong docking scores obtained for DAT suggest that mesembrine-type alkaloids may influence dopamine reuptake mechanisms, which could partly explain the reversal of locomotor deficits observed in zebrafish larvae treated with Zembrin^®^.

Furthermore, the docking results indicate interactions with the Adenosine A2A receptor, with binding energies reaching approximately −8.5 kcal/mol (Table 1). Adenosine A2A receptors are highly expressed in the striatum and play a crucial modulatory role in dopaminergic signalling pathways [38]. Antagonism of these receptors has emerged as an effective therapeutic strategy for improving motor symptoms in PD, as demonstrated by the clinical use of Istradefylline [39].

Moderate binding affinities were also observed with the dopamine D2 receptor, a key receptor involved in dopaminergic neurotransmission and motor regulation within the basal ganglia circuitry (Table 1). Dysregulation of D2 receptor signalling contributes significantly to the motor symptoms of PD, and many pharmacological treatments target this receptor to restore dopaminergic balance [37]. Interaction with this receptor further supports the possibility that mesembrine-type alkaloids may influence multiple components of dopaminergic signalling pathways.

Additionally, the binding affinities of the alkaloids toward the NMDA, 5-HT2C, and 5-HT7 receptors were moderate when compared with those observed for MAO-B, DAT, and adenosine A2A receptors (Table 1). This pattern may suggest a potential modulatory role of the alkaloids at these targets; however, further computational and experimental investigations are required to substantiate these observations.

Overall, among the predicted targets, MAO-B, DAT, and the adenosine A2A receptor appear to be the most promising due to their favourable docking scores and their well-established roles in the pathophysiology of PD. These proteins are closely involved in regulating dopamine metabolism, synaptic dopamine availability, and basal ganglia signalling. Their interaction with mesembrine-type alkaloids, therefore, suggests that compounds from *M. tortuosum* may exert therapeutic effects through a multitarget mechanism that influences dopaminergic signalling and neuroprotection. The computational results provide additional mechanistic insight into the neuroprotective and neurorestorative effects observed in the zebrafish PD model and highlight potential upstream molecular targets that may contribute to the biological activity of *M. tortuosum*. However, further experimental studies will be necessary to validate these predicted interactions and confirm their functional relevance in PD.

### 2.4. Stability Profiles of M. tortuosum Alkaloids Against MAO-B and DAT Targets

**Figure 3:**
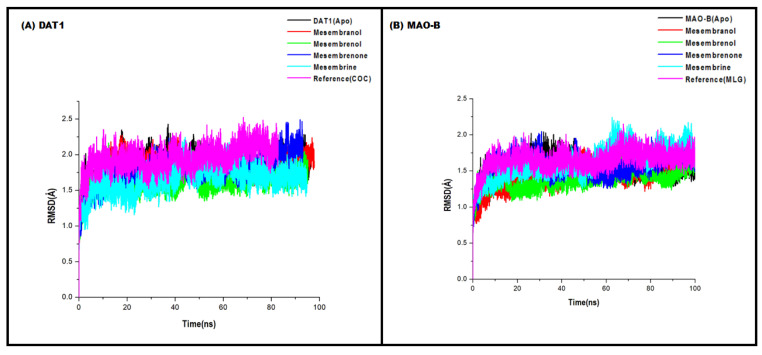
Time-dependent RMSD of DAT (**A**) and MAO-B (**B**) Cα atoms over 100 ns molecular dynamics simulations. Complexes include mesembrine-type alkaloids, reference ligands (COC for DAT and MLG for MAO-B), and apo proteins. All alkaloids reduce DAT drift relative to apo and maintain stable MAO-B binding, with final RMSD values of 2.8–3.4 Å for DAT and 0.8–0.9 Å for MAO-B, comparable to reference compounds.

Time-dependent root-mean-square deviation (RMSD) trajectories were computed for the four alkaloids mesembranol, mesembrenol, mesembrenone, and mesembrine, and the reference compounds against monoamine oxidase B (MAO-B) and dopamine transporter (DAT), both implicated in PD pathophysiology. The RMSD values, averaged over the simulation time (0–100 ns), provide a quantitative measure of ligand–target complex stability (Figure 3). For DAT, the apo protein exhibited a steady rise in the RMSD from 1.8 Å at 0 ns to 3.6 Å at 45 ns, indicating inherent conformational drift. Among the alkaloids, mesembranol, mesembrenol, mesembrenone, and mesembrine all showed very similar RMSD profiles against DAT, increasing from ~1.8 Å to ~3.4 Å over 45 ns, closely matching the reference compound (COC, RMSD ~3.4 Å at 45 ns). This suggests that these alkaloids form complexes with DAT of comparable stability to a known DAT ligand, though RMSD alone does not confirm binding affinity. Slightly lower final RMSD values (~3.2–3.3 Å) were observed for mesembranol and mesembrenol relative to mesembrine (~3.4 Å), hinting at marginally tighter binding modes.

For MAO-B, the apo protein displayed remarkably low RMSDs (starting at 1.6 Å and decreasing to 0.7 Å by 45 ns), indicative of a highly stable, rigid structure in the absence of ligands, consistent with crystallographic studies of MAO-B [40]. All four alkaloids and the reference (MLG) produced RMSD values that declined over time, from ~1.6 Å to ~0.8–0.9 Å at 45 ns, essentially matching the apo protein. Such low RMSD values, often below 1.0 Å, imply that the alkaloids do not induce significant conformational changes in MAO-B, possibly binding in a surface-exposed or already preformed pocket without destabilising the protein backbone. This behaviour is typical of reversible, competitive inhibitors that occupy the substrate cavity without large-scale rearrangements [41]. Notably, all compounds performed equally well, suggesting that the core mesembrine skeleton is sufficient for MAO-B recognition.

From a comparative perspective, the alkaloids show similar RMSD trends across both targets, but the implications differ: low and decreasing RMSDs for MAO-B point to stable, non-disruptive binding, while moderate and increasing RMSDs for DAT reflect expected flexibility in a transporter protein upon ligand engagement [42]. The absence of RMSD spikes above 3.5 Å for any alkaloid–target pair indicates no ligand dissociation events during the simulation.

Taken together, mesembrine-type alkaloids found in Zembrin^®^, mesembranol, mesembrenol, mesembrenone, and mesembrine, demonstrate highly stable binding to MAO-B, with RMSD profiles indistinguishable from the rigid apo enzyme, supporting their potential as reversible MAO-B inhibitors. For DAT, the alkaloids produce RMSD trajectories comparable to a reference ligand, suggesting moderate and stable interactions without pronounced destabilisation. Overall, the four mesembrine-type alkaloids found in Zembrin^®^ show a dual-target engagement: favourable, low-fluctuation binding to MAO-B and acceptable, controlled binding to DAT.

### 2.5. Structural Flexibility Profiles of M. tortuosum Alkaloids Against MAO-B and DAT

**Figure 4:**
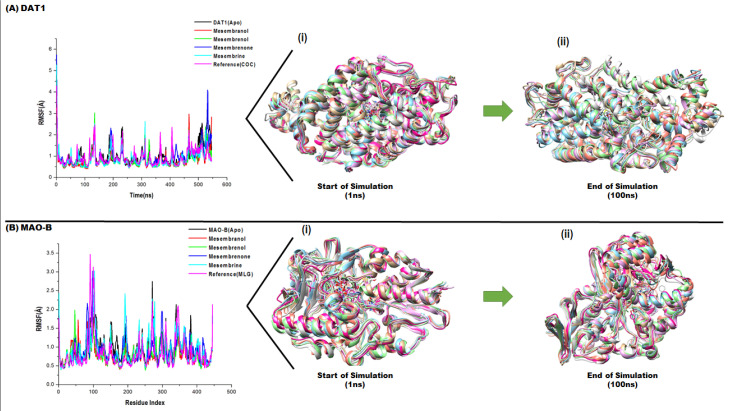
Root mean square fluctuation (RMSF) profiles of protein backbone residues over the course of the simulations for the apo form and ligand-bound complexes for DAT (**A**) and MAO-B (**B**). The plots illustrate residue-level flexibility, with peaks corresponding to regions of higher atomic fluctuation. Comparative analysis reveals changes in protein dynamics upon ligand binding, where reduced fluctuations in specific regions indicate enhanced structural stability, while increased flexibility in loop or terminal regions reflects localised conformational adaptability.

The root mean square fluctuation (RMSF) per residue was calculated for the DAT and MAO-B targets in complex with the four alkaloids alongside reference ligands (cocaine, COC, for DAT and mofegiline, MLG, for MAO-B) and the respective apo proteins (Figure 4). RMSF values provide a residue-level measure of protein backbone flexibility upon ligand binding. Lower RMSF generally indicates rigid and enhanced binding stability, whereas elevated RMSF may suggest local destabilisation or incomplete binding modes.

For DAT, the apo protein exhibited an average RMSF of 1.01 Å. All alkaloids found in Zembrin^®^ reduced the overall protein flexibility (Figure 4). Mesembranol yielded the most pronounced stabilisation with an RMSF of 0.84 Å, followed closely by mesembrenol and mesembrine at 0.86 Å. Mesembrenone showed moderate stabilisation at 0.98 Å, comparable to the reference COC (0.92 Å). The consistent lowering of the RMSF by the mesembrine alkaloids suggests that the mesembrine core stabilises DAT, likely by engaging key residues in the transporter’s substrate binding pocket without inducing excessive conformational entropy, a favourable property for long-lasting transporter modulation [42].

For MAO-B, the apoprotein displayed a baseline RMSF of 0.91 Å. Mesembranol again provided the most pronounced stabilisation (0.80 Å), followed by mesembrenol (0.82 Å) and the reference MLG (0.83 Å) (Figure 4). Mesembrenone produced RMSF values equal to the apo protein (0.91 Å), suggesting neutral effects on backbone dynamics (Figure 4). Mesembrine showed a slightly elevated RMSF of 0.90 Å, still within the baseline range. The ability of mesembranol and mesembrenol to rigidify MAO-B below its apo level is notable, as MAO-B is already a rigid enzyme [40]. This further reduction implies that these alkaloids induce additional packing interactions, likely within the substrate cavity or the entrance loop regions, without causing steric clashes. The absence of destabilisation (no RMSF values exceeding the apo baseline by >0.1 Å) across all of the tested alkaloids reinforces that mesembrine alkaloids bind MAO-B reversibly and without inducing harmful conformational changes.

Comparatively, the alkaloids generally stabilised both DAT and MAO-B, but the effect was more uniform for MAO-B across all compounds. For DAT, natural alkaloids outperformed the reference COC in reducing the RMSF. These RMSF data complement the previously reported RMSD trajectories: low RMSDs coupled with reduced RMSFs indicate that the alkaloids maintain stable overall complex geometry. This low RMSF is also evident, as indicated at the start of the simulation at 1 ns to the end of the simulation at 100 ns, where minimal structural flexibility was observed against both targets (Figure 4).

The overall impact of Zembrin^®^ is, therefore, a coordinated, favourable interaction with both targets: reduced conformational entropy at the binding sites, no evidence of induced unfolding, and in several cases, super-apo stabilisation. These computational results warrant further in vitro evaluation of mesembrine alkaloids as dual-target lead compounds for PD, with mesembranol emerging as the most promising candidate based on RMSF metrics.

### 2.6. Binding Free Energy Analysis

The computed binding free energies (ΔE_bind_) for mesembrine-type alkaloids against MAO-B and DAT are fully consistent with the RMSD and RMSF profiles, reinforcing the conclusion that these natural compounds form stable, energetically favourable complexes with both PD-relevant targets. For MAO-B, all alkaloids exhibited negative ΔE_bind_ values ranging from −34.84 kcal/mol (mesembrine) to −38.83 kcal/mol (mesembranol), comparable to the reference MLG (−38.99 kcal/mol) (Table 2). Mesembranol showed the strongest binding, which aligns with its lowest RMSF (0.80 Å) and stable RMSD trajectory (Table 2). This explains its elevated RMSF in DAT (1.19 Å) and confirms that deuteration disrupts optimal charge complementarity. For DAT, all of the alkaloids produced strong negative ΔE_bind_ values (−45.51 to −53.60 kcal/mol), superior to the reference COC (−46.14 kcal/mol). Mesembranol again ranks best (−53.60 kcal/mol), corresponding to its lowest RMSF (0.84 Å) and favourable RMSD stabilisation (Table 2). The binding is driven by highly negative electrostatic (ΔE_ele_) and van der Waals (ΔE_vdw_) terms, partially offset by positive solvation energies. In summary, the free energy analysis quantitatively validates the dynamics data: mesembranol and mesembrenol are the most potent dual stabilisers. The overall alkaloid profile of Zembrin^®^ shows consistently stronger binding to DAT than MAO-B, yet both targets are engaged with energies comparable to or superior to the reference ligand.

## 3. Discussion

Over the years, the utilisation of reserpine for in vivo modelling of PD has significantly declined, as neurotoxin-based PD models have gained prominence. Several studies have indicated that neurotoxin-based PD models, in contrast to reserpine-based PD models, offer rapid neurodegeneration [43]. Although reserpine does not induce rapid neurodegeneration, this study provides evidence that reserpine-based PD models can recapitulate both motor and non-motor symptoms associated with PD in zebrafish larvae.

In 2017, Puttonen and colleagues discovered that zebrafish larvae (4 dpf) treated with reserpine (40 µg/mL) exhibited significant locomotor deficits, particularly notable 24 h post-treatment, with no recovery observed after 72 h. Furthermore, Puttonen et al. [44] concluded that reserpine induces locomotor deficits and substantially decreases the neurotransmitters dopamine, norepinephrine, and serotonin in zebrafish larvae. Additionally, Wang et al. [24] reported a loss of dopaminergic neurons in reserpine-treated larvae, affirming reserpine’s suitability as a model for PD. Recently, Luo et al. [45] further noted that reserpine adversely affects swim behaviours and nerve signalling by inhibiting neurotransmitter release through VMAT2 inhibition [45]. This phenomenon is comparable to that observed in 6-OHDA-treated larvae reported by Feng et al. [46], suggesting that there may be potential overlap in the neurotoxic pathways of 6-OHDA and reserpine. Another study revealed that reserpine disrupts the hypothalamic–pituitary–thyroid axis and impairs thyroid hormone production, underscoring the extensive implications of PD beyond the central nervous system [47]. Furthermore, reserpine inhibits VMAT2, resulting in a significant reduction of monoamine availability in the brain, which is associated with hypolocomotion linked to depression, anxiety, and PD [48]. In 2016, Coetzee and colleagues demonstrated that Trimesemine^TM^, an extract of *M. tortuosum* comprising high content mesembrine (>70%), commercially available, enhances the upregulation of VMAT2 expression in rat hippocampus cells. The authors determined that Trimesemine^TM^ is involved in the storage and release of monoamine neurotransmitters [49]. This action is demonstrated in our investigation, as Zembrin^®^ ameliorated reserpine-induced locomotor impairments, likely by restoring monoamine neurotransmitters in the brain. Moreover, the ratio of mesembrine alkaloids in Zembrin^®^ appears to be effective, as Zembrin^®^ successfully ameliorated locomotor deficits comparable with the positive control of L-dopa. Although spontaneous locomotor activity is frequently monitored in zebrafish larvae studies using reserpine, this should be considered a behavioural consequence of monoamine depletion. The ability of L-dopa to rescue these motor deficits further highlights the involvement of dopaminergic neurotransmission and its relevance to PD. Therefore, the present findings should be interpreted within the context of monoaminergic dysfunction relevant to PD.

The various extracts of *M. tortuosum* have been investigated for antidepressant effects and neuroprotective effects, indicating potential benefit to PD. In 2024, Gericke and colleagues evaluated the antidepressant effect of Zembrin^®^ and its individual isolated mesembrine alkaloids [15]. This was achieved by treating zebrafish larvae at 5 dpf with 2 µg/L reserpine for 24 h. In the latter study, the reserpine-treated larvae had significantly decreased basal and stimulated locomotion. Furthermore, in contrast to the individual mesembrine alkaloids, it was shown that 25 µg/mL of Zembrin^®^ restored locomotor deficits caused by reserpine in zebrafish larvae (5 dpf). The study concluded that mesembrine alkaloids may have synergistic effects for antidepressant efficacy [15], but are not beneficial when used individually. In our study, Zembrin^®^ (12.5–50 µg/mL) significantly reversed reserpine-induced locomotor deficits. Furthermore, it should be noted that *M. tortuosum* extracts do not only rescue locomotor deficits in PD or depression studies. Maphanga et al. [50] found that the aqueous extract of *M. tortuosum* displayed an anxiolytic effect in zebrafish larvae light–dark locomotion model of anxiety and reverse-thigmotaxis behaviour [50]. Additionally, larvae treated with an aqueous extract from *M. tortuosum* displayed an increase in time spent in the central arena, indicative of anxiolytic activity [50]. In a follow-up study, the authors further investigated the effect of individual mesembrine alkaloids (mesembrine, mesembrenone, mesembranol, and mesembrenol) on the light–dark locomotion model of anxiety, also in zebrafish larvae (5 dpf). Although all four alkaloids (10–50 µM) seemed to increase the distance moved in the central arena, mesembrenol and mesembrenone displayed superior activity in the light–dark locomotion model of anxiety [32]. Maphanga and colleagues used in silico techniques, which suggested the mesembrine alkaloids had strong affinity for the SERT and that the anxiolytic action might be due to the SERT [32]. In 2024, Gericke et al. [15] performed a similar experiment investigating the effect of mesembrine alkaloids in a light–dark locomotion model of anxiety, and the study concluded that Zembrin^®^ (12.5–25 µg/mL) and mesembrine showed anxiolytic activity [15]. The concentrations of mesembrine alkaloids tested in the anxiolytic assays by Maphanga et al. [32] and Gericke et al. [15] differed significantly. While Maphanga et al. [32] specified concentrations ranging from 10 µM to 50 µM, Gericke and colleagues employed concentrations corresponding to the Zembrin^®^ formulation, resulting in very low concentrations that were not explicitly detailed due to proprietary considerations [15].

Oxidative stress plays a significant role in the pathogenesis of PD. To fully elucidate the mechanism of action of Zembrin^®^*,* it is necessary to examine the targets and compare them to the effects observed in this study and the potential benefits in PD. Furthermore, by inhibiting VMAT2, reserpine induces downstream oxidative stress [51,52]. The main source of ROS is produced by mitochondrial dysfunction and the excessive metabolism of monoamine neurotransmitters by the action of MAO-B. Additionally, ROS are generated when dopamine undergoes autooxidation, resulting in the formation of dopamine–quinones, reactive molecules that contribute to neurotoxicity observed in PD. The aforementioned ROS subsequently surpass the neutralising capacity of GSH, leading to the depletion of GSH evident in PD [51,52]. Therefore, it is possible that Zembrin^®^ could possess a three-point target mechanism in the modulation of reserpine-induced oxidative stress. Firstly, it was observed that selegiline, a MAO-B inhibitor, reduced ROS levels to approximately baseline levels without causing subsequent increases in tGSH. This, taken together with in silico MAO-B inhibition, suggests reserpine-induced ROS generation predominantly involves the metabolism of monoamine neurotransmitters by MAO-B. Furthermore, Coetzee et al. [49] and Luo et al. [19] indicated that Trimesemine™ and *M. tortuosum* fractions containing mesembrine, mesembrenone, and D^7^-mesembrenone exhibit moderate inhibition of MAO-B, which consequently prevents the degradation of monoamine neurotransmitters into reactive metabolites [19,49]. The second mechanism may involve a direct increase in tGSH, as demonstrated in the current study, which could subsequently mitigate the excessive ROS production in reserpine-induced oxidative stress. Finally, various extracts of *M. tortuosum* have been reported to exhibit direct ROS scavenging activity in the DPPH assay [18,53,54].

An additional mechanism of action of Zembrin^®^ is the inhibition of SERT, where the K_is_ for mesembrine, mesembrenone, and mesembrenol are 1.4, 27, and 63 nM, respectively [11,33,55]. These effects were confirmed by in silico techniques, which suggested the mesembrine alkaloids have a strong affinity for the SERT [32]. In PD, neurodegeneration affects serotonin regulation, leading to cognitive impairments and depression. The inhibition of SERT increases serotonin availability at synapses, potentially alleviating both motor and non-motor symptoms of PD. Furthermore, SERT inhibitors are used to treat depression and anxiety in PD patients [56,57].

The docking of mesembrine alkaloids against seven PD targets identified MAO-B and DAT as the most promising targets, with scores comparable to known inhibitors (selegiline and modafinil derivatives, respectively). Subsequent 100 ns molecular dynamics simulations showed stable complexes for all mesembrine alkaloids against MAO-B and for DAT. The MM/GBSA binding free energies confirm these findings with DAT binding electrostatically driven, whereas MAO-B binding relies on van der Waals interactions.

When compared with the literature, the binding energies and dynamic profiles of mesembrine alkaloids against MAO-B are similar to those reported for known reversible MAO-B inhibitors, such as safinamide and rasagiline (ΔG ≈ −30 to −40 kcal/mol in MM/GBSA studies) [58,59]. For DAT, the observed stabilisation and strong electrostatics resemble those of benztropine analogues, which are atypical dopamine reuptake inhibitors with lower abuse liability than cocaine [60]. Therefore, mesembrine alkaloids may offer a unique profile: MAO-B inhibition with neuroprotective potential, coupled with moderate DAT interaction that could alleviate both the motor and non-motor symptoms of PD without inducing psychostimulation. Furthermore, Zembrin^®^ (12.5 µg/mL) displayed good activity in the locomotor and oxidative stress assays, while mesembrine-type alkaloids contained in Zembrin^®^ indicated dual activity in MAO-B and DAT molecular docking and dynamics assessments.

## 4. Materials and Methods

### 4.1. Chemicals and Reagents

The following reagents were obtained from Sigma Aldrich (Johannesburg, South Africa): 2′,7′-dichlorofluorescein diacetate (H2DCFDA), anhydrous magnesium chloride, bicinchoninic acid (BCA), ammonia, dichloromethane (AR grade), anhydrous calcium chloride, anhydrous sodium sulphate, dimethyl sulfoxide (DMSO), anhydrous magnesium sulphate, L-dopa, HEPES sodium salt, methanol, selegiline, sulphuric acid, sodium sulphate, sodium chloride, sucrose, potassium chloride, and phenylmethylsulphonyl fluoride (PMSF). Zembrin^®^ was supplied by HG&H Pharmaceuticals (Johannesburg, South Africa).

### 4.2. Plant Material Sourcing and Extraction

#### 4.2.1. Zembrin^®^ Preparation

Zembrin^®^ was provided in the form of a dry powder, which was reconstituted and stored in DMSO before diluting with 0.3× Danieau media, in accordance with the appropriate concentrations required for the analysis.

#### 4.2.2. Phytochemical Profiling Using Ultra-Performance Liquid Chromatography-Mass Spectrometry (UPLC-MS) Analysis

For UPLC-MS analysis, 1 mg of Zembrin^®^ (Batch no: SCE0419-1402) dissolved in 1 mL of methanol was filtered and injected into a Waters Acquity UPLC system (Waters, Milford, MA, USA). The analysis was performed using the method previously developed in our laboratory [61]. Briefly, the method involved using a BEH C18 column (150 mm × 2.1 mm, i.d., 1.7 μm particle size, Waters, Milford, USA) at 30 °C, with a mobile phase of 0.1% ammonium hydroxide and 90% acetonitrile at a flow rate of 0.3 mL/min. Gradient elution proceeded from 80% A to 60% A in 2 min, and to 50% A in 4.5 min, returning to the initial ratio in 0.2 min. The total run time was 8.5 min, with the data analysed through Masslynx 4.1^®^ software. Mass spectrometry used positive electrospray ionisation with nitrogen as the desolvation gas at 500 L/h and a desolvation temperature of 350 °C, collecting data in the *m*/*z* range of 100–1000.

### 4.3. Zebrafish Husbandry and Embryo Production

Wild-type laboratory-bred adult zebrafish were housed in polycarbonate tanks at a density of five adult fish per litre of reverse osmosis water in a ZebTec Active Blue standalone self-regulating aquatic system (Tecniplast, Varese, Italy). The water parameters were monitored and maintained at pH 6.8–7.5, conductivity 400–800 µS, temperature 26–28.5 °C, ammonia < 0.02 mg/L, nitrates < 50 mg/L, nitrites < 0.1, and a 14/10 h light/dark cycle, in accordance with standards of zebrafish care [62]. The fish were fed three times a day with dry feed in the morning and at midday, as well as artemia (high-protein live feed) in the late afternoon. The fish selected for spawning were placed in breeding tanks at a ratio of 1:2 (female:male), separated with a spacer and kept overnight in the dark. At the end of the dark cycle the following morning, the spacers were removed, and the fish were allowed to mate for 1 h. Embryos were collected and placed in a Petri dish, suspended in 0.3× Danieau medium (5 mM NaCl, 0.17 mM KCl, 0.33 mM CaCl_2_, 0.33 mM MgSO_4_, and 1.5 mM HEPES buffer at pH 7.2), and observed under a microscope (ZEISS, Oberkochen, Germany) to remove unfertilised eggs. The healthy embryos were incubated at 28 °C, and medium changes were performed every 24 h until the larvae reached the required experimental age of 4 dpf.

### 4.4. Parkinson’s Disease Model Using Reserpine

**Figure 5:**
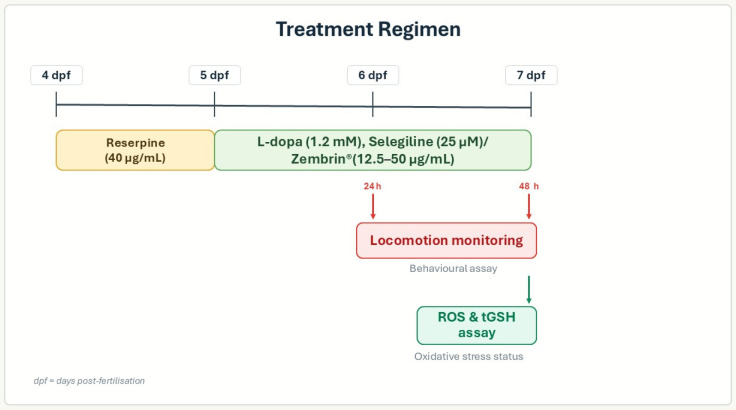
The treatment regimen for the induction and assessment of a PD model.

To assess the effects of Zembrin^®^, the treatment regimen followed the method of Puttonen et al. [44], with modifications. Selegiline (25 µM) and L-dopa (1.2 mM) were used as positive controls, and the untreated control received 0.3× Danieau medium. Zebrafish larvae at 4 dpf were pre-treated with reserpine (40 µg/mL) for 24 h, followed by exposure to various concentrations of Zembrin^®^ (12.5, 25.0 and 50.0 µg/mL), selegiline (25 µM), and L-dopa (1.2 mM) for a further 48 h at 28 °C (Figure 5). Fresh drug treatments were changed daily until the end of the experiment. This study selected Zembrin^®^ concentrations based on preliminary MTC assessments in zebrafish larvae at 4 dpf. Ten larvae were allocated to each Zembrin^®^ concentration (100, 250, and 500 µg/mL), and daily monitoring for mortality and anomalies was performed until 7 dpf.

### 4.5. Monitoring Locomotion

In the locomotion assay, six larvae per treatment group were individually placed in a 48-well plate. The experiment was independently repeated three times, resulting in a total of 18 larvae per group. Locomotor activity was monitored in a temperature-controlled DanioVision (Noldus, Wageningen, The Netherlands) observation chamber equipped with Noldus EthoVision^®^ XT software version 15 (Noldus, Wageningen, The Netherlands). Initially, the larvae were allowed 5 min to acclimatise; thereafter, locomotion was monitored as total distance travelled (mm) over a period of 10 min.

### 4.6. Oxidative Stress Status

#### 4.6.1. Reactive Oxygen Species (ROS)

The ROS were quantified following the method described by Lackmann et al. [63]. At the end of the treatment period, 15 larvae per treatment group were pooled and humanely euthanised in an ice slurry. They were then rinsed in 0.3× Danieau medium and incubated in 5.0 μM of H_2_DCFDA fluorescent probe for 45 min at 28 °C in the dark. After incubation, the larvae were rinsed in 0.3× Danieau medium to remove excess dye. The larvae were euthanised in ice slurry for 20 min and subsequently transferred to Eppendorf tubes, where excess liquid was removed. The larvae were homogenised in ice-cold 60 μL extraction buffer (HEPES buffer, 320 mM sucrose, 0.1 mM MgCl_2_, and 0.5 mM PMSF at pH 7.4) using a pellet pestle. The homogenates were subsequently centrifuged at 13,000 rpm and 4 °C for 20 min. After centrifugation, 10 μL aliquots of supernatant were added to white opaque 96-well plates in triplicate, and an additional 10 μL was added for protein quantification, using the bicinchoninic acid (BCA) method. A volume of 150 μL of the buffer (30 mM HEPES buffer, 200 mM KCl, and 1 mM MgCl_2_) was added to each well, and the plates were covered in aluminium foil and placed on a shaker for 10 min. The fluorescent intensity of each well was measured on a SpectraMax M2 microplate reader (Molecular Device, San Jose, CA, USA) at an excitation wavelength of 485 nm and an emission wavelength of 515 nm. Reactive oxygen species levels were expressed as arbitrary fluorescent units per mg of protein.

#### 4.6.2. Total Glutathione Content Determination (tGSH)

At the end of the incubation period, 15 larvae per treatment were rinsed with 0.3× Danieau medium in Petri dishes. The larvae were euthanised in an ice slurry, homogenised in 100 μL extraction buffer, and mixed with 150 μL of 5% sulfosalicylic acid. The homogenate was centrifuged at 13,000 rpm for 10 min at 4 °C, and the supernatant was collected to measure the total glutathione content (tGSH) using a glutathione kit (Sigma-Aldrich, St. Louis, MO, USA). Total glutathione levels were measured at an emission wavelength of 412 nm on a SpectraMax M2 microplate reader. An additional 10 μL of each supernatant was used for protein quantification.

### 4.7. In Silico Analysis

#### 4.7.1. Biological Target Identification

To identify potential biological targets associated with PD, a combined approach integrating literature-based evidence and computational target prediction tools was employed. Initially, an extensive review of the published literature was conducted to compile receptors and signalling pathways known to play a role in the pathogenesis of PD, including those involved in dopaminergic dysfunction, monoamine neurobiology, and oxidative stress. Furthermore, SwissTargetPrediction was used to predict probable molecular targets for the mesembrine alkaloids based on their chemical structures. The predicted targets were then cross-referenced with PD-related targets reported in the literature to identify biologically relevant overlaps. Attention was given to targets that are known to participate in agonistic or antagonistic interactions, as well as those involved in enzymatic inhibition or receptor modulation, that could be of therapeutic value in the management of PD.

#### 4.7.2. Molecular Docking

The X-ray crystal structures of the selected target proteins were retrieved from the RCSB Protein Data Bank, including monoamine oxidase B (MAO-B; PDB ID: 2BXR), dopamine D2 receptor (PDB ID: 7DFP), N-methyl-D-aspartate receptor (PDB ID: 7EOT), adenosine A2A receptor (PDB ID: 8RW0), dopamine transporter (DAT; PDB ID: 9EO4), serotonin 5-HT2C receptor (PDB ID: 8DPF), and serotonin 5-HT7 receptor (PDB ID: 7XTC). All protein structures were prepared using the Dock Prep module in UCSF Chimera version 1.12. Preparation steps included the removal of co-crystallised ligands, ions, and water molecules, followed by the addition of missing hydrogen atoms, assignment of partial charges, and correction of incomplete side chains where necessary. The prepared structures were subsequently saved in PDBQT format for docking.

The three-dimensional structures of the mesembrine-type alkaloids (mesembranol, mesembrenol, mesembrine, and mesembrenone) were obtained from PubChem and geometry-optimised using Avogadro to obtain energetically stable conformations. Gasteiger charges were assigned, and the ligands were saved in PDBQT format.

Molecular docking simulations were carried out using the AutoDock Vina version 1.1.2 plugin integrated within Chimera. Grid boxes were defined around the active sites of each protein, guided by the position of co-crystallised ligands. The grid dimensions were adjusted to ensure adequate coverage of the binding pocket, while allowing sufficient space for ligand flexibility. Docking parameters were set to default values, with an exhaustiveness level sufficient to ensure reliable sampling of ligand conformations.

For each ligand–protein pair, multiple binding poses were generated, and the best-ranked conformations were selected based on the lowest binding affinity (kcal/mol) and favourable interaction profiles. These analyses were used to assess the binding potential of the alkaloids and to identify key residues contributing to ligand stabilisation within the target proteins. To validate the docking protocol, redocking of the co-crystallised ligands was performed with RMSD < 2 Å considered acceptable, as shown in the Supplementary Table S3.

#### 4.7.3. Molecular Dynamics Simulation

Molecular dynamics simulations (MDSs) were carried out for two of the targets to which the compounds bound favourably after docking, namely, MAO-B and DAT receptors. Molecular dynamics simulations are necessary to validate docking results and provide a more robust assessment of the binding energy of all complexes over time. Using UCSF Chimera, hydrogen atoms were removed, and AMBER.ff14SB charges were assigned to the compounds, which were then saved individually in .mol2 file format, while the protein structures were saved in .pdb format. Subsequently, all MDSs were carried out using the graphics processing unit (GPU) version of the Particle Mesh Ewald Molecular Dynamics (PMEMD) in the AMBER 18 software package [64]. The ANTECHAMBER module was used to assign atomic partial charges to the compounds, applying the Restrained Electrostatic Potential (RESP) method along with the General AMBER Force Field (GAFF) protocol [65]. The FF14SB in the Amber 18 suite was used to parametrise the protein [66]. The Link Edit and parm (LEaP) module in Amber 18 was then used to add missing hydrogen and heavy atoms. Depending on the complex, LEaP was also used to neutralise the systems by adding Na^+^ and Cl^−^ counter ions and solvating them in a TIP3P water box with a 10 Å buffer. LEaP was further used to generate the input and topology files for downstream processing [67]. To eliminate unfavourable contacts during the molecular dynamics (MDs) simulations, each system underwent energy minimisation for 2000 steps. This involved an initial 1000 steps using the steepest descent method, followed by 1000 steps with the conjugate gradient algorithm, both performed without restraints. The systems were then gradually heated from 0 K to 300 K under an NPT ensemble, using a harmonic restraint of 5 kcal/mol·Å^2^ and a Langevin thermostat with a collision frequency of 1 ps^−1^. All systems were then equilibrated at 300 K for 500 ps without restraints, maintaining constant pressure at 1 bar using the Berendsen barostat. The SHAKE algorithm was applied to constrain all bonds involving hydrogen atoms [68]. A 100 ns unrestrained MD production run was then carried out for each system, using a target temperature coupling time of 2 ps and maintaining constant pressure at 1 bar. Analysis of the trajectories and coordinates generated from the MD run was carried out using the CPPTRAJ and PTRAJ modules [69] incorporated in Amber 18. The binding free energy was then estimated.

#### 4.7.4. Binding Free Energy Analysis Using the Molecular Mechanics/Generalised Born Surface Area (MM/GBSA) Method

The binding free energy of each ligand complex was estimated using the MM/GBSA method [70].

Mathematically, the binding free energy (ΔG_bind_) is calculated as follows:

ΔG_bind_ = G_complex_ − G_receptor_ − G_ligand_ (1)

ΔG_bind_ = E_gas_ + ΔG_sol_ − TS (2)

where ΔG_bind_ is the summation of the gas phase and solvation energy terms less the entropy (TS) term.

E_gas_ = E_int_ + E_vdw_ + E_elec_ (3)

E_gas_ is the sum of the AMBER force field internal energy terms E_int_ in the internal energy (bond, angle, and torsion), the covalent van der Waals (E_vdw_) and the non-bonded electrostatic energy component (E_elec_).

The solvation energy is calculated from the following equation:

G_sol_ = G_GB_ + G_non-polar_ (4)

G_non_polar_ = γSASA + b (5)

where ΔG_bind_ is taken as the sum of the gas phase and solvation energy terms less the entropy (TΔS) term, and G_complex_ represents the energy of the receptor ligand complex, while G_receptor_ and G_ligand_ represent energies of the receptor and ligand, respectively. E_gas_ denotes gas-phase energy; E_int_ signifies internal energy; and E_ele_ and E_vdw_ indicate the electrostatic and van der Waals contributions, respectively. E_gas_ is the gas phase, elevated directly from the FF14SB force terms. G_sol_ denotes solvation free energy, which can be decomposed into polar and nonpolar contribution states. The polar solvation contribution, G_GB_, is determined by solving the GB equation, whereas G_SA_, the nonpolar solvation contribution, is estimated from the solvent accessible surface area (SASA), determined using a water probe radius of 1.4 Å. T and S, corresponding to temperature and total solute entropy, respectively, and γ is a constant [69].

### 4.8. Data Analysis

The data were presented as the mean ± standard error of the mean values of triplicate experiments. The data were analysed using the non-parametric Kruskal–Wallis test, and group comparisons were determined by Dunn’s post hoc test. A *p*-value < 0.05 was considered statistically significant, and all statistical analyses were performed using GraphPad Prism version 8.0 (USA).

## 5. Conclusions

Molecular docking, molecular dynamics simulations, and binding free energy calculations collectively demonstrate that mesembranol, mesembrenol, mesembrenone, and mesembrine form stable, energetically favourable complexes with both MAO-B and DAT, two key targets implicated in PD. This balanced dual engagement distinguishes the mesembrine alkaloid mixture from many synthetic agents that target only a single pathway. Taken together, the integrated experimental and computational results indicate that Zembrin^®^ holds promise for alleviating PD by reducing oxidative stress, normalising locomotor function, and dually modulating MAO-B and DAT. Although this study’s limitation is its focus on monoaminergic transmission induced by reserpine, particularly oxidative stress and locomotion, which does not fully encompass the multifactorial nature of PD, it provides a foundation for future research on mesembrine alkaloids in the treatment of PD. Further studies could explore extra-monoaminergic effects, including neuroinflammation, neurotrophic factors, and neurotransmitters relevant to PD.

## Figures and Tables

**Table 1:** Molecular docking of mesembrine-type alkaloids against selected PD targets.

Targets	Binding Score (Kcal/mol)				
	Mesembranol	Mesembrenol	Mesembrenone	Mesembrine	Positive
Monoamine oxidase MAO-B	−8.7	−8.5	−8.7	−8.3	MLG = −6.8
Dopamine transporter (DAT)	−8.1	−8.1	−8.7	−8.4	COC = −9.0
N-methyl-D-aspartate receptor	−6.8	−6.7	−7.0	−6.8	J86 = −7.3
Adenosine A (2A) receptor	−8.2	−8.1	−8.5	−8.4	JQ9 = −8.9
D2 receptor	−7.4	−7.8	−7.8	−8.1	SIP = −11.3
Serotonin 2C (5-HT2C)	−7.5	−7.4	−7.7	−7.3	T4U = −6.2
Serotonin 7 (5-HT7)	−7.4	−7.3	−7.5	−7.9	8K3 = −7.3

**Table 2:** Binding free energy results of mesembrine alkaloids against DAT and MAO-B targets.

System	Energy Contributions (Kcal/mol)				
	ΔE_vdw_	ΔE_ele_	ΔE_gas_	ΔE_sol_	ΔE_bind_
MAO-B					
Mesembranol	−43.74 ± 0.36	116.98 ± 1.20	73.25 ± 1.26	−112.07 ± 1.13	−38.83 ± 0.42
Mesembrenol	−40.66 ± 0.36	117.87 ± 1.93	77.22 ± 2.08	−112.79 ± 1.80	−35.58 ± 0.44
Mesembrenone	−41.77 ± 0.24	108.27 ± 1.56	66.50 ± 1.58	−103.69 ± 1.32	−37.19 ± 0.84
Mesembrine	−37.93 ± 0.27	107.61 ± 2.28	69.68 ± 2.27	−104.51 ± 1.95	−34.84 ± 0.47
Reference (MLG)	−39.89 ± 0.25	94.23 ± 1.41	54.34 ± 1.54	−93.32 ± 1.31	−38.99 ± 0.39
DAT					
Mesembranol	−51.03 ± 0.27	−120.62 ± 0.88	−171.65 ± 0.87	118.06 ± 0.78	−53.60 ± 0.35
Mesembrenol	−48.55 ± 0.26	−133.83 ± 0.89	−182.39 ± 0.89	131.33 ± 0.81	−51.05 ± 0.43
Mesembrenone	−46.25 ± 0.31	−128.06 ± 0.96	−174.32 ± 0.98	128.80 ± 0.98	−45.51 ± 0.65
Mesembrine	−46.26 ± 0.28	−127.87 ± 1.02	−174.13 ± 0.92	127.17 ± 0.92	−46.96 ± 0.35
Reference (CoC)	−48.74 ± 0.39	−136.11 ± 1.33	−184.85 ± 1.30	138.70 ± 1.13	−46.14 ± 0.38

## Data Availability

The original contributions presented in this study are included in the article. Further inquiries can be directed to the corresponding authors.

## Supplementary Materials

The following supporting information can be downloaded at: https://www.mdpi.com/article/10.3390/molecules31132369/s1. Table S1: Gridbox coordinates; Table S2: Potential targets for mesembrine alkaloids; Table S3: Docking protocol validation-Redocking RMSD values for the cocrystalised ligands of selected targets.

## Abbreviations

The following abbreviations are used in this manuscript:

AREC	Animal research ethics committee
H2DCFDA	2′,7′-dichlorofluorescein diacetate
BBB	Blood–brain barrier
BCA	Bicinchoninic acid
DAT	Dopamine transporter
dpf	Days post-fertilisation
DMSO	Dimethyl sulfoxide
DPPH	2,2-diphenyl-1-picrylhydrazyl
hpf	Hours post-fertilisation
MAO-B	Monoamine oxidase-B
NPIs	Non-pharmacological interventions
PD	Parkinson’s disease
PMSF	Phenylmethylsulphonyl fluoride
ROS	Reactive oxygen species
SERT	Serotonin re-uptake transporter
tGSH	Total glutathione content
VMAT2	Vesicular monoamine transporter 2
