# Supplementary material for: Zembrin® Mitigates Reserpine-Induced Motor Dysfunction and Oxidative Stress in Parkinson’s Disease: In Vivo and In Silico Analyses
Source: Molecules. 2026 Jul 5;31(13):2369. doi: 10.3390/molecules31132369 (PMC13362728; doi:10.3390/molecules31132369)
Supplement: Supplementary file 1 [file molecules-31-02369-s001.zip › molecules-4384804-supplementary.pdf]

Zembrin® Mitigates Reserpine-Induced Motor Dysfunction and Oxidative Stress in Parkinson's Disease: In Vivo and In Silico Analyses

Keagile Lepule [1], Maxleene Sandasi [1,2], Elliasu Salifu [1] and Alvaro Viljoen [1,2,*]

[1] Department of Pharmaceutical Sciences, Tshwane University of Technology, Private Bag X680, Pretoria 0001, South Africa, keagilebasetsana@gmail.com (K.L.); sandasim@tut.ac.za (M.S.); salifuey@tut.ac.za (E.S.)

[2] SAMRC Herbal Drugs Research Unit, Faculty of Science, Tshwane University of Technology, Pretoria 0001, South Africa

* Correspondence: viljoenam@tut.ac.za; Tel.: +27-12-382-6360

Supplementary Data

Supplementary Table S1. Gridbox Cordinates

**Target**	**Gridbox**	**X**	**Y**	**Z**
**MAO-B**	Centre	19.66	3.43	6.02
	Dimensions	24.56	22.48	19.18
**DAT1**	Centre	131.44	124.82	124.48
	Dimensions	26.76	22.84	19.18
**NMDA**	Centre	138.20	114.33	131.40
	Dimensions	21.65	22.48	25.16
**Adenosine**	Centre	0.07	1.33	122.84
	Dimensions	16.73	26.39	19.37
**D2 receptor**	Centre	-94.00	-20.55	214.77
	Dimensions	24.85	24.39	27.30
**5-HT2C**	Centre	129.74	144.61	102.03
	Dimensions	20.72	18.85	16.70
**5-HT7**	Centre	88.66	110.80	82.34
	Dimensions	22.61	19.81	19.53

Supplementary Table S2. Potential targets for mesembrine alkaloids

Name of potential target	Type	Function	Reference	Compound Prediction (SwissTargetPrediction)
**Monoamine oxidase MAO-B**	Inhibition	Inhibition of dopamine metabolism could result in improved locomotion and oxidative stress.	https://doi.org/10.1016/B978-0-323-79680-4.00021-8.	Mesembrine, Mesembranol, Mesembrenol, Mesembrenone,
**Dopamine transporter (DAT)**	Inhibition	Inhibition could increase dopamine levels at the synaptic cleft could improve locomotion	https://doi.org/10.1124/jpet.116.232371	Mesembrine, Mesembranol, Mesembrenol, Mesembrenone,
**N-methyl-D-aspartate receptor**	Antagonist	A dopamine deficiency results in overactive excitatory pathway, which can be blocked NMDA antagonists, to restore a more balanced neurotransmission in the motor circuit.	https://doi.org/10.1038/s41398-018-0162-2	D7-Mesembrenone, Mesembrenol, Mesembrenone.
**Adenosine A (2A) receptor**	Antagonist	Blockade of A2A-receptor enhances the activity of the D2-receptors	https://doi.org/10.1212/01.wnl.0000073136.00548.d4	Mesembranol, Mesembrenol, Mesembrenone,
**D2 receptor**	Agonist	Mimics postsynaptic effects of dopamine on D2 receptor	https://doi.org/10.1016/B978-0-323-79680-4.00021-8.	Mesembrine, Mesembranol, Mesembrenol, Mesembrenone
**Serotonin 5-HT2C receptor**	Inhibition	A G-protein-coupled receptor primarily located in the central nervous system that acts as a "neural rheostat" regulating mood, appetite, anxiety, and neurobiological network excitability.	https://doi.org/10.1016/j.ejphar.2007.04.030	Mesembrine, Mesembranol, Mesembrenol, Mesembrenone,
**Serotonin 5-HT7 receptor**	Inhibition	a G-protein coupled receptor (GPCR) that regulates essential neurophysiological and physiological processes, including circadian rhythms, sleep, cognition (learning and memory), mood regulation, and thermoregulation.	https://link.springer.com/article/10.1007/s00221-013-3694-y	Mesembrine, Mesembranol, Mesembrenol, Mesembrenone

Supplementary Table S3. Docking protocol validation-Redocking RMSD values for the co-crystalised ligands of selected targets

Target	PDB	Co-crystallized ligand	Dock score	Redocking RMSD (Å)	Acceptable (< 2.0 Å)
**MAO-B**	2BXR	MLG	-6.8	0.63	✓
**DAT1**	9EO4	COC	-8.4	1.08	✓
**NMDA**	7EOT	J86	-7.3	1.78	✓
**Adenosine**	8RW0	JQ9	-8.9	1.23	✓
**D2 receptor**	7DFP	SIP	-11.3	1.95	✓
**5-HT2C**	8DPF	T4U	-6.2	1.46	✓
**5-HT7**	7XTC	8K3	-7.3	1.88	✓
